# A Case Report of Acute Necrotizing Pancreatitis after COVID-19 Infection

**DOI:** 10.1155/2022/3258677

**Published:** 2022-07-29

**Authors:** Amir Mohammad Salehi, Hossain Salehi, Maryam Hasanzarrini, Ensiyeh Jenabi, Aida Alizamir

**Affiliations:** ^1^School of Medicine, Hamadan University of Medical Sciences, Hamadan, Iran; ^2^Gastroenterology Ward, Farheekhtegan Hospital, Islamic Azad University of Medical Sciences, Tehran, Iran; ^3^Clinical Research Development Unit of Shahid Beheshti Hospital, Hamadan University of Medical Science, Hamadan, Iran; ^4^Autism Spectrum Disorders Research Center, Hamadan University of Medical Sciences, Hamadan, Iran; ^5^Department of Pathology, School of Medicine, Fatemieh Hospital, Hamadan University of Medical Sciences, Hamadan, Iran

## Abstract

Coronavirus 2019 (COVID-19), which is associated with many systemic inflammatory reactions and high morbidity and mortality rates, became a serious public health problem and led to a rapid epidemic. Fever, dry cough, and shortness of breath are the most common symptoms of COVID-19. In addition to respiratory symptoms, gastrointestinal manifestations of COVID-19 are increasingly known to progress more rapidly than other symptoms and can occur in cases of mild infection or even after remission of the viral infection. Acute pancreatitis (AP) caused by COVID-19 is one of the rare gastrointestinal symptoms which is an acute inflammatory disease of the pancreas that is associated with high complications. Here, we report acute COVID-19-induced pancreatitis in a 38-year-old man who died.

## 1. Introduction

Coronavirus 2019 (COVID-19), is an ongoing global epidemic that has affected millions of people and has become a serious public health problem [1]. Although respiratory symptoms, including fever, dry cough, and shortness of breath, are the most common, gastrointestinal (GI) manifestations of COVID-19 are also increasingly recognized, with diarrhea being the most common manifestations of GI [2, 3].

Acute pancreatitis (AP) is a common disease, being the gastrointestinal disease most frequently requiring acute hospitalization [4]. Viral infections are the most common cause of acute pancreatitis. Other common causes are gallstones and alcohol abuse [5]. Multiple kinds of viruses including cytomegalovirus, Coxsackie B virus, Hepatitis A, B (Hep A, B), echovirus, Epstein–Barr virus, enterovirus, and human immunodeficiency virus (HIV) are known to cause AP [6]. With the advent of the COVID-19 epidemic, several cases of AP have been reported following infection with SARS-CoV-2. Herein, we report a case of AP with recently diagnosed COVID-19 infection without other risk factors for pancreatitis.

## 2. Case Presentation

**Figure 1:**
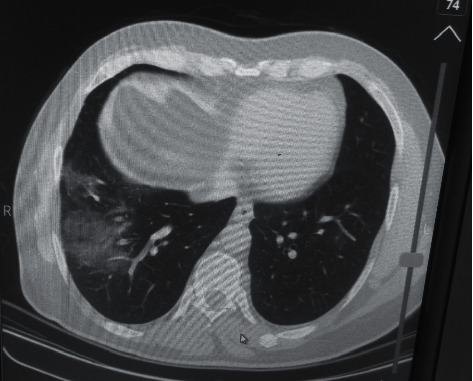
Lung CT scan of the patient at previous admission shows moderate involvement of the patient's lungs.

A 38-year-old male with a chief complaint of nausea and severe epigastric pain with radiation to the back area was referred to the emergency room in Hamedan hospital. The patient was admitted to the hospital one week ago due to COVID-19 pneumonia with significant lung involvement (85 O_2_ saturation (Sat)) (Figure 1), so medical therapy with remdesivir (200 mg stat dose and then 100 mg Q12 hr) and dexamethasone (8 mg Q12 hr) was received just in hospital admission. The patient during hospital admission improved and was discharged without any complications and medications, but one week after discharge from the hospital, he suddenly developed severe epigastric pain that radiated to the back area and nausea, so he has referred to the hospital again.

**Figure 2:**
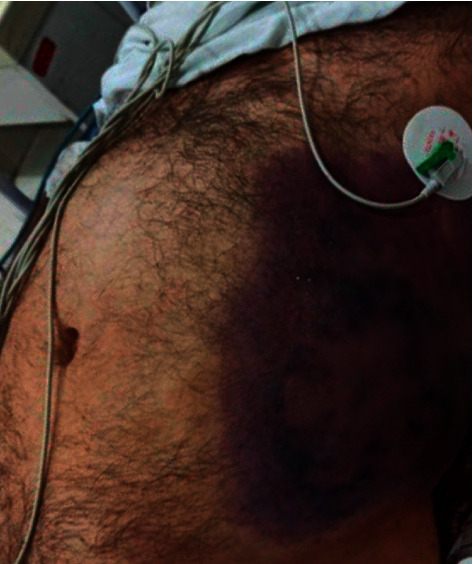
Ecchymosis in the left flank consistent with Grey Turner's sign.

Past and personal history was normal. In the physical exam, the temperature was 38 C, respiratory rate (RR) 30/min, heart rate (HR) 115/min, blood pressure (BP) 95/65 mm Hg, 95Sat, the mucosa was dry, jugular venous pressure was normal, and no lymphadenopathy and thyromegaly, but in the chest, examination decreased breathing sound was heard. In the abdominal examination, generalized tenderness and distention and one area of ecchymosis in the left flank were seen (Figure 2). So, with the impression of pancreatitis, he was admitted and some workup was performed (Table 1).

**Figure 3:**
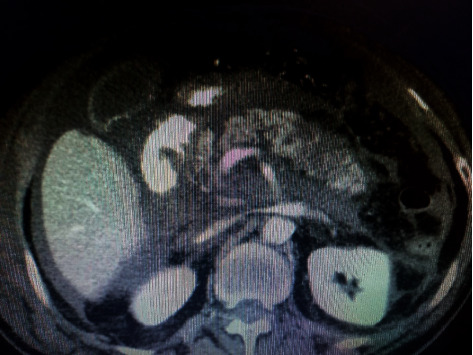
Computed tomography scan of the abdomen showing necrotic areas (hypoenhancing, nonenhancing) in the body of the pancreas.

Sonography showed severe interstitial pancreatitis with suspicion of necrotizing pancreatitis, so a spiral abdominal CT scan with the pancreatic protocol was performed favoring severe necrotizing pancreatitis (>30%) peripancreatic fluid accumulation (Figure 3). Fluid therapy with ringer lactate and antibiotic therapy (meropenem 500 mg Q12 h) was started, but the patient gradually developed a decreased level of consciousness and shock state and decreased O_2_ saturation. We did not have the opportunity for surgery to resect the necrotic tissues because the patient was in shock and did not respond despite supportive treatment, and the patient's condition did not stabilize. The patient underwent mechanical ventilation and supportive care, but his condition deteriorated and expired after one day.

## 3. Discussion

AP is an acute inflammatory disease of the pancreas associated with high morbidity. Vomiting, nausea, and abdominal pain are common presentations of acute pancreatitis. It can be diagnosed by the presence of epigastric pain and tenderness to palpitation and abnormal increase of pancreatic enzymes (amylase or lipase) more than the three-time above the normal limit and/or imaging criteria (computed tomography, magnetic resonance imaging, and ultrasound) [7]. In our patient, AP presented with epigastric pain and nausea, and laboratory findings and imaging confirmed the diagnosis of AP. We were also able to exclude potential causes of AP, including alcoholism, drugs, hypertriglyceridemia, hypercalcemia, Hep B and HIV infection, previous attacks or family history, and trauma, based on the patient's history and tests.

Previously, Müller et al. isolated the SARS-CoV-2 nucleocapsid protein in beta cells and pancreatic exocrine cells in postmortem patients and found that SARS-CoV-2 could infect and proliferate in the pancreatic islets of the Langerhans Islands and cause infection-related diabetes [8]. Although ACE2 and TMPRSS2 receptors, which are involved in virus binding and penetration, express more in pancreatic outlet cells than in pancreatic islet cells [9], for SARS-CoV-2, it is unclear whether tissue damage leading to AP occurs as a result of direct SARS-CoV-2 infection [10] or as a result of systemic multiple organ dysfunction syndrome (MODS) with increased levels of lipase which leads to the breakdown of triacylglycerols in adipose tissue cells and the formation of cytokine storms as a result of the toxic effect of unsaturated fatty acids on mitochondria [11] or following coagulation cascade activation caused by active inflammatory process due to SARS-CoV-2 infection. In this case, AP is associated with increased fibrinolysis resulting in higher levels of D-dimers [12].

The patient's condition after an AP attack is directly related to the severity of the pancreatitis SIRS reaction. One of the scaling systems for the severity of pancreatitis at the onset of symptoms is the Ranson scoring system, which is clinically efficient and useful [6]. It is evaluated using clinical and laboratory variables at the time of patient referral to the hospital. Five variables are measured at admission (age, WBC count, glucose, LDH, and AST) and six variables are measured within 48 hours (Hct drop, BUN increase, calcium, arterial pO2, base deficit, and, fluid sequestration). A Ranson score of 0 or 1 predicts that complications will not develop and that mortality will be negligible [13]. A score of 3 or greater predicts severe AP and possible mortality [13]. In our patient, there were 5 variables of Ranson's criteria (Table 2). It should be noted that serum amylase or lipase levels are only indicative of acinar cell damage and have nothing to do with the severity of pancreatitis or the likelihood of complications [14].

CT scan is the gold standard for definitive diagnosis of AP and also provides prognostic information to the physician. Baltazar et al. developed a grading system based on CT scan findings that included 5 grades: A (normal pancreas), B (enlargement of pancreas), C (inflammatory changes in pancreas and peripancreatic fat), D (ill-defined single peripancreatic fluid collection), and E (two or more poorly defined peripancreatic fluid collections), that our patient was in grade E [6]. It is important to understand that the severity of AP is not fixed. Therefore, a CT scan at any point in time does not accurately reflect the severity of the disease at another point in time, and in some cases, such as AP caused by SIRS, a series of CT scans are needed to control and monitor the course of the disease [15].

To reduce pancreatic secretion, discontinue oral feeding until pain and tenderness resolve and serum amylase and white blood cell count (WBC) return to normal. In severe cases such as our patient, several liters of the isotonic solution are needed to resuscitate the patient, so maintaining adequate tissue perfusion by monitoring hemodynamic parameters and maintaining adequate intravascular volume is very important. Therefore, electrolytes and blood glucose need direct control. Various studies have also shown that all pharmacological efforts to reduce pancreatic secretion, including anticholinergic drugs, somatostatin analogs, antacids, and aprotinin, have no significant benefit [16, 17].

In acute pancreatitis, respiratory function is carefully monitored by pulse oximetry because fluid accumulation within the pleural space and atelectasis interferes with the rise of the respiratory diaphragm. In some cases, the use of a ventilator is also required [6].

## Figures and Tables

**Table 1:** Laboratory results on admission.

Test	Results	Reference range
White cell count (per mm^3^)	18500	4000–11,000
Total bilirubin (mg/dl)	1	0.1–1.2
Direct bilirubin (mg/dl)	0.22	Less than 0.3
Lactate dehydrogenase (U/L)	341	140–280
Blood urea nitrogen (mg/dl)	39	7–20
Hematocrit (%)	33.8	36.7–46.4
Creatinine (*μ*mol/L)	1.07	0.8–1.2
Amylase (U/L)	773	30–110
Lipase (U/L)	286	0–160
Triglycerides (mg/dl)	119	Less than 150
Total cholesterol (mg/dl)	221	Less than 200
C-reactive protein	Positive	+/-
Random blood glucose (mg/dl)	151	80–140
Hepatitis A virus serology	Negative	
Hepatitis B virus serology	Negative	
Hepatitis C virus serology	Negative	
Human immunodeficiency virus serology	Negative	

**Table 2:** Ranson criteria for our patient.

Criteria	Our patient
On admission	
WBC > 16k	+(18500)
Age > 55	—
Glucose > 200 mg/dL (>10 mmol/L)	−(151)
AST > 250	+(350)
LDH > 350	−(341)
48 hours into admission	
Hct drop > 10% from admission	—
BUN increase>5 mg/dL (>1.79 mmol/L) from admission	+(28)
Ca <8 mg/dL (<2 mmol/L) within 48 hours	+(1.4 mmol/L)
Arterial pO_2_ < 60 mmHg within 48 hours	+(58 mmHg)
Base deficit (24-HCO_3_) > 4 mg/dL within 48 hours	—
Fluid needs > 6L within 48 hours	−(2.5)

## Data Availability

Access to data is possible with permission from the responsible author.
